# The development of a HAMstring InjuRy (HAMIR) index to mitigate injury risk through innovative imaging, biomechanics, and data analytics: protocol for an observational cohort study

**DOI:** 10.1186/s13102-022-00520-3

**Published:** 2022-07-15

**Authors:** Bryan C. Heiderscheit, Silvia S. Blemker, David Opar, Mikel R. Stiffler-Joachim, Asheesh Bedi, Joseph Hart, Brett Mortensen, Stephanie A. Kliethermes, The HAMIR Study Group, Geoffrey Baer, Craig Buckley, Kyle Costigan, Shauna Drew, Duffy Eberhardt, Kurrel Fabian, Herman Feller, Erin Hammer, Danielle Heidt, Kenneth Lee, Brian Lund, Jack Martin, Michael Moll, Jennifer Sanfilippo, Shaun Snee, Claire Tanaka, Ty Taylor, John Wilson, Devin Woodhouse, Yi-Chung Lin, Jack Hickey, Nirav Maniar, Frances Taylor, Ryan Timmins, Matthew Cousins, Olivia DuCharme, Xue Feng, Scott Magargee, Craig Meyer, Anthony Nguyen, Lara Riem, Robin West, Steven Allen, Dain Allred, Anthony Beutler, Dustin Bruening, Darren Campbell, A. Wayne Johnson, Camille Nguyen, Emma Remington, Annie A. Smedley, Joshua K. Sponbeck, David Berkoff, Josh Berkowitz, Thomas Birchmeier, Troy Blackburn, Malvika Choudhari, Mario Ciocca, Alessa Lennon, Caroline Lisee, Noah McCoy, David Mincberg, Scott Oliaro, Brian Pietrosimone, Luke Ross, Julie Titter, Sarah Sund

**Affiliations:** 1 Badger Athletic Performance Program, Department of Orthopedics and Rehabilitation, University of Wisconsin-Madison, 1685 Highland Avenue, 6136 Medical Foundation Centennial Building, Madison, WI 53705 USA; 2 Springbok Analytics, Charlottesville, VA USA; 3 Sports Performance, Recovery, Injury and New Technologies Research Centre, School of Behavioural and Health Sciences, Australian Catholic University, Fitzroy, VIC Australia; 4 NorthShore Orthopedic and Spine Institute, Skokie, IL USA; 5 University of North Carolina-Chapel Hill, Chapel Hill, NC USA; 6 Brigham Young University, Provo, UT USA; 7 American Medical Society for Sports Medicine Collaborative Research Network, Leawood, KS USA

**Keywords:** Muscle injury, Risk prediction model, Sprint running, Muscle volume, Eccentric strength

## Abstract

**Background:**

The etiology of hamstring strain injury (HSI) in American football is multi-factorial and understanding these risk factors is paramount to developing predictive models and guiding prevention and rehabilitation strategies. Many player-games are lost due to the lack of a clear understanding of risk factors and the absence of effective methods to minimize re-injury. This paper describes the protocol that will be followed to develop the HAMstring InjuRy (HAMIR) index risk prediction models for HSI and re-injury based on morphological, architectural, biomechanical and clinical factors in National Collegiate Athletic Association Division I collegiate football players.

**Methods:**

A 3-year, prospective study will be conducted involving collegiate football student-athletes at four institutions. Enrolled participants will complete preseason assessments of eccentric hamstring strength, on-field sprinting biomechanics and muscle–tendon volumes using magnetic-resonance imaging (MRI). Athletic trainers will monitor injuries and exposure for the duration of the study. Participants who sustain an HSI will undergo a clinical assessment at the time of injury along with MRI examinations. Following completion of structured rehabilitation and return to unrestricted sport participation, clinical assessments, MRI examinations and sprinting biomechanics will be repeated. Injury recurrence will be monitored through a 6-month follow-up period. HAMIR index prediction models for index HSI injury and re-injury will be constructed.

**Discussion:**

The most appropriate strategies for reducing risk of HSI are likely multi-factorial and depend on risk factors unique to each athlete. This study will be the largest-of-its-kind (1200 player-years) to gather detailed information on index and recurrent HSI, and will be the first study to simultaneously investigate the effect of morphological, biomechanical and clinical variables on risk of HSI in collegiate football athletes. The quantitative HAMIR index will be formulated to identify an athlete’s propensity for HSI, and more importantly, identify targets for injury mitigation, thereby reducing the global burden of HSI in high-level American football players.

*Trial Registration* The trial is prospectively registered on ClinicalTrials.gov (NCT05343052; April 22, 2022).

## Background

The incidence and burden of hamstring strain injury (HSI) is high in a number of sports involving high-speed running, including American football [1]. Understanding factors that increase the risk of HSI in football players is paramount to developing predictive models and guiding prevention strategies. The etiology of HSI is multi-factorial, with eccentric hamstring strength receiving particular attention as a primary modifiable risk factor. However, recent research suggests that eccentric hamstring strength is only weakly associated with future occurrence of HSI [2] and that the use of eccentric hamstring strength as a variable to predict future HSI is limited [3].

Hamstring strain injuries typically occur when the muscle–tendon unit (MTU) is exposed to some combination of (1) high forces; (2) lengthening action; (3) high velocity; (4) moderate-to-long length [4–8]. This current understanding of the injury mechanism suggests both the structure and function of the hamstrings require consideration to appropriately model an athlete’s risk of future HSI. Structurally, a larger ratio of hamstring muscle width to aponeurosis width (i.e. a large muscle relative to a smaller aponeurosis) results in higher levels of tissue strain at the muscle–tendon junction (the most common site of HSI) during running [9]. Hamstring function during running is a key element to consider when assessing HSI risk. During the second half of the swing phase, the hamstrings are active, rapidly lengthening and absorbing energy to decelerate the limb in preparation for foot contact [4]. Hamstring muscle force increases approximately 1.3-fold as running velocity increases from 80 to 100% of maximum and the greatest MTU stretch is incurred by the biceps femoris long head during high-speed running [10]. As such, “poor” running mechanics that increase MTU strain have long been considered a causative factor for HSI; however, there is limited empirical data to indicate if running mechanics influence HSI risk [11, 12].

Despite identification of important structural characteristics in the etiology of HSIs, the translation of these measures into practice has been limited due, in part, to the time and effort required to obtain them. That is, the assessment of muscle and tendon morphology requires manual segmentation of many “slices” of MRIs taken along the length of the thigh, a laborious and time-consuming process. Developing intuitive solutions to increase the adoption of measures of hamstring muscle and tendon morphology in practice is of critical importance for improving HSI prediction models. Similarly, the assessment of running mechanics is currently restricted to laboratory-based environments, which limits its practical utility. The advent of wearable sensors, such as inertial measurement units (IMUs), has the potential to allow for field-based measures of running biomechanics to assist with HSI risk determination. Developing a validated, field-based solution will lead to an increased capability to assess running mechanics and its association with future HSI.

While much attention has focused on identifying factors associated with first-time HSI, the rates of recurrent HSI are high [13, 14]. The impact of a prior HSI on structural and functional characteristics of the hamstrings, which may subsequently predispose to recurrent injury, is largely overlooked. Long-term deficits exist in musculotendon characteristics following HSI including: biceps femoris long head atrophy with compensatory short head hypertrophy; reduced muscle quality with fatty infiltration; and an increase in tendon cross sectional area due to scar tissue rather than tendon-specific hypertrophy [15–18]. These factors contribute to an increase in muscle strain under eccentric loading adjacent to the site of the index injury [17]. Determining the relevance of these changes for modeling risk of recurrent injury is of paramount importance given that prior injury is the most consistently identified risk factor for future HSI [19].

This current study aims to combine quantitative imaging, on-field biomechanics, and computational analytics into the largest-of-its-kind study of elite collegiate football players to develop the HAMstring InjuRy (HAMIR) index that can be used to reduce the burden of HSI by identifying potential future targets for HSI risk mitigation and prophylactic approaches. This paper describes the protocol that will be followed to achieve the following specific aims:

1. Develop a risk prediction model for HSI based on morphological, architectural, biomechanical and clinical factors in National Collegiate Athletic Association (NCAA) Division I (D1) collegiate football players.
2. Develop a risk prediction model for recurrent HSI based on morphological, architectural, biomechanical and clinical factors (identified in Aim 1) in NCAA D1 collegiate football players.

## Methods/design

### Overall study design and study sites

**Fig. 1:**
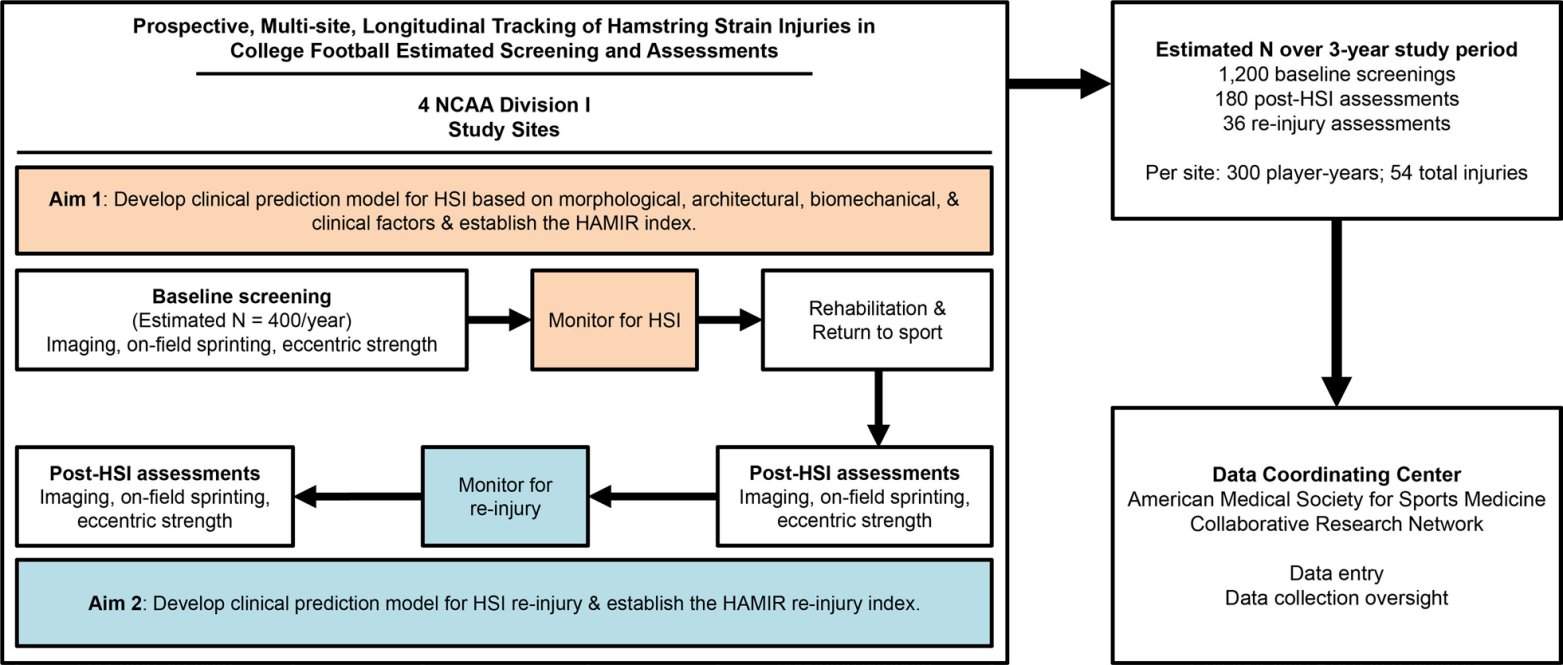
Overall design of the 3-year HAMIR study. NCAA, National Collegiate Athletic Association

This is a longitudinal, prospective study of NCAA D1 collegiate football student-athletes across four institutions (Fig. 1). The University of Wisconsin-Madison, Brigham Young University, and University of North Carolina-Chapel Hill will begin study enrollment in the spring of 2022 and will end data collection following the completion of the spring football season in 2025. The fourth site will be added in fall of 2022 and begin study enrollment in spring of 2023 with data collection anticipated to end following the spring football season in 2026.

**Fig. 2:**
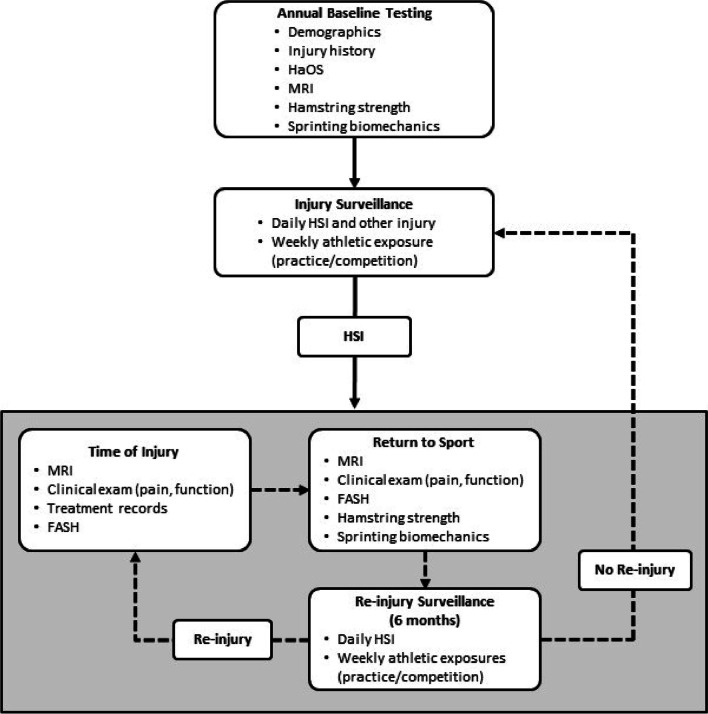
Annual injury tracking and data collection process. HaOS, Hamstring Outcome Score; FASH, Functional Assessment Scale for Acute Hamstring Injuries

The overall study flow for each year is depicted in Fig. 2. All enrolled participants will complete preseason evaluations of eccentric hamstring strength along with an IMU-based assessment of sprinting biomechanics and conventional magnetic-resonance imaging (MRI). Athletic trainers will monitor injuries as well as practice and competition participation (i.e. exposure) for the duration of the study (including in- and off-season training). Participants who sustain an HSI at any point during the study will undergo a clinical assessment at the time of injury along with MRI examinations. Following completion of structured rehabilitation and return to unrestricted sport participation, clinical assessments, MRI examinations and sprinting biomechanics will be repeated. Injury recurrence will be monitored from time of injury clearance for return to sport through a 6-month follow-up window.

### Data coordinating center

The American Medical Society for Sports Medicine’s Collaborative Research Network (AMSSM CRN) will serve as the data coordinating center (DCC) for this study. The DCC is responsible for providing administrative and regulatory support to all study sites, data management, monitoring and analysis, and overseeing quality assurance of the data provided by each site. During the study planning phase, the DCC assisted in development of the centralized web data capture platform, using the Research Electronic Database Capture (REDCap) system [20, 21] and a manual of operating procedures to ensure consistency and reproducibility of study policies and procedures across study sites. The DCC will lead the training of each site on data entry into REDCap as well as study policy and protocol contained in the manual of operating procedures prior to initiating study enrollment.

The AMSSM CRN is experienced in facilitating multi-site investigations and is additionally responsible for leading communication across sites as it relates to data management and study protocol. Regular meetings between the DCC and site study coordinators will be established and regular reports will be provided from the DCC to the study sites to ensure accurate data capture. The lead biostatistician within the DCC (SAK) will also work closely with the study principal investigators to oversee all statistical analyses for the HAMIR study group.

### Participant recruitment and eligibility

Prior to athlete recruitment and enrollment, the study will be approved by the Institutional Review Boards of all participating institutions with all participants providing written informed consent. All rostered members of each site’s football team will be solicited to participate during each year of the 3-year study. Primary recruitment will occur prior to the fall season of each study year, from April through August. Additional recruitment for newly enrolled athletes and those who were less than 18 years old at the last enrollment opportunity will occur prior to the spring football seasons of each study year.

Study personnel at each site will meet with athletic trainers, physicians, and other key medical staff to inform them about the study. Additionally, study personnel will attend preseason meetings to recruit and consent participants. Participants will be asked to consent for the entire period of time during the study period in which they are eligible to participate. Eligibility criteria include student-athletes between the ages of 18 and 26 years of age and those rostered on the varsity football team of each participating institution during the study period. Exclusion criteria will include history of malignant disease and known contraindications to MRI.

### Injury outcomes and athlete exposures

The primary outcomes for this study include index HSI and HSI re-injury. An index HSI will be defined as an acute injury to the posterior thigh that limits full, unrestricted sports participation and includes two or more of the following symptoms: palpable pain along the hamstring muscles, posterior thigh pain without radicular symptoms, weakness and/or pain with resisted knee flexion, and pain with running. HSI re-injury will be defined as an acute HSI that occurs to either limb within 6 months of an index HSI. Team athletic trainers will record daily individual HSI reports for each participant throughout the study, including in-season and off-season training. Athletic trainers will also record the occurrence and time away from sport for the following injuries: lower extremity injuries that require surgery (e.g. anterior cruciate ligament rupture; hip labral tear or femoroacetabular impingement, athletic pubalgia, tendon ruptures), other strains (e.g. gastrocsoleus complex, quadriceps, adductors) and concussions. Athletic trainers will complete weekly team reports that include the number of practice and competition exposures for each participant. An injury risk exposure will be defined as one athlete participating in one practice or competition.

### MRI protocol and analysis

An MRI examination of the lumbo-pelvic region and bilateral lower extremities will be performed on all participants prior to the start of preseason (baseline). This MRI examination will be performed using 3 T scanners, a flexible surface coil, and will consist of a lumbo-pelvic and bilateral lower extremity scan to quantify muscle volumes within the entire field of view, as well as a bilateral thigh-specific scan to provide detailed contrast of the tendons and aponeuroses. The full lower extremity scan parameters were defined to create high muscle signal and low fat signal. Depending on the study site, it will consist of either an axial multi-slice 2D gradient echo fast SPGR sequence (TR/TE: 850 ms/4.5 ms, FOV 500 mm × 375 mm, 256 × 192 matrix, 5 mm thick, with fat suppression) or an axial 3D T1 DIXON sequence (TR/TE: 3.88 ms/1.1 ms and 2.2 ms, FOV 500 mm × 375 mm, 256 × 192 matrix, 5 mm thick). We have confirmed that muscle volumes calculated from each of these sequences are equivalent. Axial images will be obtained contiguously from the 12th thoracic vertebra to below the ankle joint in acquisition sets of 40 images. The tendon scan parameters were defined to highlight the contrast between tendons/aponeuroses, muscle, and fat. For all sites, it will consist of axial T1-weighted images (TR/TE: 175 ms/2.1 ms, FOV: 500 mm × 375 mm, 512 × 384 matrix, 5 mm thick, no fat suppression). The tendon scan will include axial images starting from the hamstrings’ origin on the ischium to the insertions on the tibia. MRI examinations will be repeated on those participants that sustain an HSI at the time of injury and at time of return to sport. For all sites, these examinations will include the tendon scan (as described above) as well as a fat-suppressed T2-weighted sequence (TR/TE = 7220/82.6 ms, FOV: 500 mm × 375 mm, 512 × 384 matrix, 5 mm thick) for assessment of muscle edema.

Analysis of the MR images will include quantification of all viewable muscle volumes, hamstring tendon lengths and cross-sectional areas, aponeurosis cross-sectional areas and area of attachment with muscle, scar volume and morphology, and edema volume. Automatic muscle segmentation will be performed using Springbok machine learning technology [22]. The algorithms will be updated and refined to include automatic segmentation of tendons, scar, and edema, based on training datasets that will be generated using manual segmentation. MR images obtained at time of injury will be assessed by a musculoskeletal radiologist and injury classification determined.

### Acquisition and processing of IMU data

Seven IMUs (MTw Awinda, Xsens Technologies B.V., The Netherlands) will be affixed on each participant (sacrum, left and right thigh, left and right shank, left and right foot) using adhesive tape and/or straps. Prior to data collection, calibration of the IMUs will be conducted via the proprietary manufacturer software (Xsens MVN, Xsens Technologies B.V., The Netherlands).

Following calibration, a static trial will be collected with the participant standing in the anatomical position to provide baseline data for sensor orientation. Once the static trial is completed, participants will then complete a maximal velocity over-ground sprinting trial from a standing start. Additional trials will be completed as needed after a minimum of two minutes of rest.

Hip, knee and ankle joint kinematic data across time will be extracted from the entirety of all the sprinting trials (including the acceleration and deceleration phases) in addition to horizontal velocity and acceleration of the center of mass.

### Nordbord strength testing

Eccentric hamstring strength will be measured using the NordBord Hamstring Testing System (Vald Performance, Newstead QLD, Australia) in accordance with a previously described protocol [23]. Participants will kneel on a padded platform with each ankle secured in a hook immediately superior to the lateral malleoli. Participants will be instructed to slowly lower themselves, resisting with their hamstrings, with arms across the chest and with shoulders, knees, and hips kept in a straight line. Three warm-up trials will be performed, one each at 50%, 75%, and 90% of maximal effort. Participants will then complete three trials at maximal effort with a 30 s rest after the warm-up trials and between each maximum effort trial. Trials will be accepted if the athlete maintains proper alignment during the trial, a distinct peak in maximal force output followed by a rapid decline is observed, and peaks are within 20% across the three trials. Up to two additional trials will be allowed if a distinct peak is not reached or the maximum force was more than 20% different from previous trials. Data from all trials will be recorded, and the maximum force obtained for each limb out of all trials meeting inclusion criteria will be used for subsequent analysis.

### Collection of morphological, biomechanical and clinical variables

Table 1 provides an overview of the data that will be collected at baseline, within 7 days of initial HSI and within 7 days of return to sport following HSI for each year of the study. These are described in more detail below.

**Table 1:** Data collection and corresponding timepoints

Assessment	Measurement	Assessment time^a^								
		Year 1			Year 2			Year 3		
		1	2	3	1	2	3	1	2	3
Demographics		X			X			X		
Clinical	Pain and function		X	X		X	X		X	X
	Injury history	X			X			X		
Patient reported outcomes	HaOS questionnaire [24]	X			X			X		
	FASH questionnaire [27]		X	X		X	X		X	X
Biomechanical and neuromuscular	Eccentric strength	X		X	X		X	X		X
	Sprinting biomechanics	X		X	X		X	X		X
MRI	Muscle, tendon and edema morphology	X	X	X	X	X	X	X	X	X

^a^(1) Baseline; (2) Within 7 days of initial injury; (3) Within 7 days of return to sport

HaOS, Hamstring Outcome Score; FASH, Functional Assessment Scale for Acute Hamstring Injuries

#### Baseline

At baseline, demographic and clinical characteristics including age, height, weight, playing position, prior HSI and detailed surgical history will be recorded for all participants. Additionally, participants will complete the Hamstring Outcome Score (HaOS) which is a functional patient reported outcome survey specific to their hamstring history (e.g. prior HSI) and hamstring function in training, competition and daily life [24]. Additionally, all participants will undergo baseline MRI examinations and testing of on-field sprinting biomechanics and eccentric hamstring strength. Sprinting biomechanics will be paired with MRI data to determine MTU strain. Eccentric hamstring strength of each limb will be assessed during the Nordic hamstring exercise [23, 25].

#### Time of injury and return to sport

Within 7 days of any diagnosed HSI, participants will undergo an additional MRI examination. A standard clinical examination will be conducted bilaterally to assess the location and extent of HSI and to evaluate the level of pain and stiffness with palpation, and hamstring range of motion and strength [26]. All participants with an HSI will undergo a post-injury rehabilitation program standardized by site [23]. Adherence to the rehabilitation program will be documented at all clinic sessions. Additionally, participants will complete the Functional Assessment Scale for Acute Hamstring Injuries (FASH) to measure the severity and impact of their symptoms on function and sports ability [27].

For participants who sustain an HSI, sprinting biomechanics and eccentric strength (as conducted at baseline) will be repeated at the return to sport time point. Determination of return to sport will be standardized by site. Within 7 days of the athlete being cleared for return to sport, the athlete will undergo another MRI examination. The same pain and clinical assessments performed at the time of injury, including the FASH, will be repeated [26].

### Sample size estimation

Sample size estimates were based on current recommended steps for calculating the minimum sample size necessary for prediction model development [28]. We anticipate enrolling approximately 100 football student-athletes at each site in their first year of data collection (with the opportunity for athletes to continue in the study during years 2 and 3). In years 2 and 3, we anticipate enrolling approximately 20 new student-athletes (primarily freshmen and transfers) at each site. This will result in an estimated enrollment of approximately 560 unique student-athletes after 3 years of data collection at each site and approximately 1200 player-years across all sites over the study period. Assuming, conservatively, an HSI prevalence of 15%, 5% attrition beyond graduation and transfer (e.g. season ending injuries), a within-subject correlation of 0.3 across study years, and an R^2^ (proportion of variability explained by model) of 20% we will be sufficiently powered to build an index HSI HAMIR index inclusive of approximately 10–12 candidate predictor parameters. Assuming 20% of the student-athletes who sustain an index HSI will re-injure, we conservatively estimate 180 student-athletes and 36 re-injuries will be available to develop a “re-injury” HAMIR index. Using the same modeling assumptions as for the index HSI, this will allow us to create a risk prediction model with up to 4 prediction parameters. The R package *pmsampsize* was used to estimate sample size requirements.

### Planned statistical approach

Univariate and bivariate summary statistics and distributional plots will be examined for all candidate predictor variables to assess modeling and hypothesis testing assumptions; appropriate transformations will be considered. Candidate predictors for the prediction models will include standard demographic and clinical measures, muscle strain during sprinting (mechanical), and muscle morphology (e.g. muscle, scar and edema volume). The HAMIR index prediction models for index HSI injury and re-injury will be constructed using generalized estimating equations (GEE) for binary outcomes with a logit link, and will control for athletic-exposures. The correlation among individuals in repeated years will be accounted for using an appropriately identified correlation matrix structure. Clustering at the institution level will be considered and modeled as necessary. The mechanism for missing data will be assessed during the modeling stage and multiple imputation methods will be considered if necessary. The final risk prediction model will provide predictions of the average estimated risk of HSI (or re-injury) for an athlete given the values of model variables.

We will internally validate the models using standard internal validation criteria for the development of risk prediction models. The overall performance of the models will be assessed using the Brier score and calibration assessed using the Hosmer Lemeshow Goodness of Fit Chi-square, with a value < 20 representing sufficient calibration. We will assess discrimination with the c-statistic, with a value of 0.8 or higher indicative of excellent discrimination. Bootstrapping will be used to assess the potential optimization of the model.

## Discussion

The high incidence of HSI and recurrent HSI in American football continues to burden professional leagues in regards to athlete performance and financial loss. As such, there is a need to better understand the reasons for HSI injury and re-injury in these athletes so that appropriate risk reduction strategies can be created to ultimately reduce the incidence of HSI. The most appropriate strategies for reducing risk of HSI are likely multi-factorial and depend on risk factors unique to each athlete. To our knowledge, this study will be the largest-of-its-kind (1200 player-years) to gather detailed information on index and recurrent HSI, and will be the first study to simultaneously investigate the effect of morphological, biomechanical and clinical variables on risk of HSI in elite collegiate football athletes. The quantitative HAMIR index will be formulated to identify an athlete’s propensity for HSI, and more importantly, identify targets for injury mitigation, thereby reducing the global burden of HSI in elite American football players.

Because this is strictly an observational study, we cannot guarantee the same practices will occur across all sites (e.g., rehabilitation protocols, return to sport decisions), nor can we control against major program changes such as coaching and staff turnover across the duration of the study period or differences in practice and competition schedules between sites. However, these issues represent realistic variability in clinical care and sports seasons; we intend to account for site-to-site variability and athlete exposure when developing the HAMIR index. Moreover, each site has agreed to adhere to the same protocol for detailed data collection on injury management, and all MRI and IMU data will be processed at a central location. As is common to prospective cohort studies, there is the potential that we are unable to capture all protocolized timepoints on all enrolled athletes; however, the manual of operations addresses specific policies for site coordinators to follow to minimize potential missing data. Additionally, the study analysis plan proposes to assess any missingness in data collection and will account for it, as appropriate, in the analytic phase. Our ability to create a meaningful recurrent HAMIR index is entirely dependent on the number of recurrent HSIs observed during the study period. Risk factors for re-injury are largely unknown and no objective data are available to guide practitioners on return-to-play decisions following HSI. We estimate only being able to include up to 4 predictive parameters in such an index for recurrent HSI and this may or may not be sufficient to accurately predict re-injury risk. However, this information is urgently needed by practitioners and we will minimally be able to provide a preliminary predictive model that can be further tested as more re-injury data is captured in this population.

## Data Availability

Not applicable.

## Abbreviations

HAMIR: Hamstring Injury Index
HSI: Hamstring strain injury
IMU: Inertial measurement units
MRI: Magnetic resonance imaging
MTU: Muscle–tendon unit
NCAA: National Collegiate Athletic Association
D1: Division I
REDCap: Research Electronic Data Capture
HaOS: Hamstring Outcome Score
FASH: Functional Assessment Scale for Acute Hamstring Injuries
GEE: Generalized estimating equations
DCC: Data coordinating center
